# Phase II study of TP300 in patients with advanced gastric or gastro-oesophageal junction adenocarcinoma

**DOI:** 10.1186/s12885-016-2828-6

**Published:** 2016-10-10

**Authors:** David Propper, Keith Jones, D. Alan Anthoney, Wasat Mansoor, Daniel Ford, Martin Eatock, Roshan Agarwal, Michiyasu Inatani, Tomohisa Saito, Masaichi Abe, T. R. Jeffry Evans

**Affiliations:** 1 Centre for Experimental Cancer Medicine, Barts Cancer Institute, Queen Mary University of London, Lower Ground Floor, Old Anatomy Building, Charterhouse Square, London, UK EC1M 6BQ; 2 Chugai Pharmaceuticals Europe Ltd. Turnham Green, London, W4 1NN UK; 3 St James Institute of Oncology, University of Leeds & Leeds Teaching Hospitals Trust, Leeds, LS9 7TF UK; 4 Department Medical Oncology, Christie Hospital, Wilmslow Road, Withington, Manchester, M20 4BX, UK; 5 Cancer Research Clinical Trials Team, Old Clinical Investigations Building, City Hospital, Dudley Road, Birmingham, B18 7QH UK; 6 Northern Ireland Cancer Clinical Trials Unit, Belfast City Hospital, East Podium, C Floor, Belfast, BT9 7AB UK; 7 West London Cancer Research Network, Coulter Suite, 1st Floor Mint Wing, St Mary’s Hospital, Praed St, London, W2 1 NY UK; 8 Chugai Pharmaceutical Co., Ltd, Nihonbashi Muromachi 2-1-1, Chuo-ku, Tokyo, 103-8324 Japan; 9 Beatson West of Scotland Cancer Centre, University of Glasgow, Glasgow, G12 OYN UK

**Keywords:** Topoisomerase-I inhibitor, Phase II study, Advanced Gastric or Gastro-oesophageal junction adenocarcinoma, Siewert Types II & III, Pharmacodynamics, Pharmacokinetics, Safety profile, Oesophago-gastric adenocarcinoma

## Abstract

**Background:**

TP300, a recently developed synthetic camptothecin analogue, is a highly selective topoisomerase I inhibitor. A phase I study showed good safety and tolerability. As camptothecins have proven active in oesophago-gastric adenocarcinomas, in this phase II study we assessed the efficacy and safety of TP300 in patients with gastric or gastro-oesophageal junction (GOJ) adenocarcinomas.

**Methods:**

Eligible patients had metastatic or locally advanced gastric or Siewert Types II or III GOJ inoperable adenocarcinoma. Patients were chemotherapy naïve unless this had been administered in the perioperative setting.

TP300 was administered as a 1-h intravenous infusion every 3 weeks (a cycle) for up to 6 cycles at a starting dose of 8 mg/m^2^ with intra-patient escalation to 10 mg/m^2^ from cycle 2 in the absence of dose-limiting toxicity. Tumour responses (RECIST 1.1) were assessed every 6 weeks. Toxicity was recorded by NCI-CTCAE version 3.0. Using a modified two-stage Simon design (Stage I and II), a total of 43 patients were to be included providing there were 3 of 18 patients with objective response in Stage I of the study.

**Results:**

In Stage I of the study 20 patients (14 males, 6 females), median age 67 years (range 40 − 82), performance status ECOG 0/1, with GC [14] or GOJ carcinoma [6] were enrolled. Of the 16 evaluable patients, 11 received the planned dose increase to 10 mg/m^2^ at cycle 2, 2 decreased to 6 mg/m^2^, and 3 continued on 8 mg/m^2^. There were no objective responses after 2 cycles of treatment. Twelve patients had stable disease for 1 − 5 months and 4 had progressive disease. Median progression free survival (PFS) was 4.1 months (CI [1.6 − 4.9]), median time to progression (TTP) was 2.9 months (CI [1.4 − 4.2]). Grade 3/4 toxicities (worst grade all cycles) included 7 patients (35 %) with neutropenia, 4 patients (20 %) with anaemia, 2 patients (10 %) with thrombocytopenia, and 3 patients (15 %) with fatigue.

This study was terminated at the end of Stage I due to a lack of the required (3/18) responders.

**Conclusions:**

This study of TP300 showed good drug tolerability but it failed to demonstrate sufficient efficacy as measured by radiological response.

**Trial registration:**

EU-CTR 2009-012097-12 2009-09-03

## Background

Inhibition of topoisomerase-I (Topo-1) is a clinically effective treatment strategy for many patients with cancer [1]. Irinotecan hydrochloride is the most widely used Topo-1 inhibitor with activity against a wide range of cancers (e.g. colorectal, glioma, oesophageal, gastric, non-small cell lung and pancreatic cancers), either as a single agent or in combination [2–5]. Irinotecan has, however, a number of properties that limit its usefulness. It is metabolized enzymatically by carboxylesterase 2 (CES2), predominantly within the liver, to SN-38 (a significantly more potent Topo-1 inhibitor). This conversion shows considerable inter-individual variability, resulting in a wide range of systemic SN-38 exposure for a given dose that may influence the efficacy and toxicity of irinotecan. Clinically, the use of irinotecan can be limited by diarrhoea and neutropenia, with potential impact on dose intensity, as well as patient acceptability. Low activity of the SN-38 metabolising enzyme UGT1A1 is associated with an increased risk of diarrhoea and myelosuppression [5], and in 2005 the US FDA recommended irinotecan dosing be modified in patients carrying the UGT1A1*28 polymorphism [2, 6]. The development of Topo-1 inhibitors not subject to such pharmacogenomic variability might, therefore, enhance the clinical efficacy and utility of this class of agents.

**Fig. 1:**
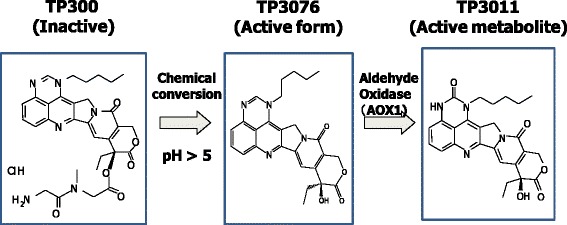
The fate of TP300, active form (TP3076) and its metabolite (TP3011)

TP300 (glycine,glycyl-N-methyl-,(9S)-9-ethyl-9,10,13,15-tetrahydro-10,13-dioxo-1-pentyl-1H,12H-pyrano[3'',4'':6',7']indolizino[2',1':5,6]pyrido[4,3,2-de]quinazolin-9-yl ester, hydrochloride) has been developed as a water soluble prodrug of the Topo-1 inhibitor TP3076, and its active metabolite, TP3011, both of which are equipotent to SN-38 in terms of Topo-1 inhibition [7] (Fig. 1). TP300 has activity at nanomolar concentrations across a range of tumour types *in vitro* and, unlike SN-38, appears active in tumours over-expressing the breast cancer resistance protein [BCRP] [7]. In man, TP300 is converted non-enzymatically to TP3076, then metabolized to TP3011 by aldehyde oxidase 1 in liver. TP3011 metabolite and TP3076 are equipotent as Topo-1 inhibitors, with an IC_50_ in the sub-nanomolar range [8]. Importantly, TP3076 lacks the phenolic-OH group required for glucuronidation so should not be influenced significantly by polymorphisms in the UGT1A1 gene. It is postulated that this will result in less variation in activation and toxicity with TP300 than with irinotecan; specifically, it would not be expected to cause severe diarrhoea.

In preclinical models TP300 has superior anti-tumour activity and a wider therapeutic index than CPT-11 in various tumour types, including gastric cancer [8]. Furthermore TP300 showed additive or synergistic antitumor effects in combination with other anti-cancer drugs such as capecitabine, oxaliplatin, cisplatin, bevacizumab and cetuximab.

A Phase I study of TP300 was performed in patients with chemotherapy insensitive or refractory tumours [9]. TP300 had predictable hematologic toxicity, and diarrhoea was uncommon. AUC at MTD was substantially greater than for SN-38. TP3076 and TP3011 are equipotent with SN-38, suggesting a pharmacokinetic advantage. Pharmacologically, it was confirmed that TP300 was 100 % transformed to its active form (TP3076) and rapidly and extensively converted to an active metabolite (TP3011). There was a linear relationship between AUC and dose, up to 10 mg/m^2^ and AUC was not affected by UGT1A1 or aldehyde oxidase genotype.

Topoisomerase I inhibitors such as irinotecan have shown activity in the treatment of gastric cancer, with single agent irinotecan demonstrating a response rate of 23 %. Phase II studies in patients with gastric cancer have demonstrated single-agent response rates of approximately 20 % for a number of chemotherapeutic agents including irinotecan [10].

Gastric cancer is one of the most common malignant diseases worldwide. The prognosis of this aggressive tumour is poor, even when detected in the early stages and treated by radical resection [11]. In most cases, metastases are already present at the time of diagnosis. Compared to purely supportive treatment, systemic chemotherapy leads to a significant extension of survival and clear improvement in the quality of life with respect to the reduction of tumour-related symptoms. This was demonstrated in various randomized studies [12–18].

However, objective remissions are often only short-term, most patients die of the disease within 1 year and response to chemotherapy does not always translate into improvement in survival. The tumours respond to treatment, but quickly become resistant. It is therefore necessary to search for new substances and more effective therapies are clearly needed for patients with metastatic gastric cancer.

Several drugs, when used as single agents, have been associated with a reduction of more than 50 % in measurable tumour mass (i.e. an “objective response” in over 15 % of patients). Topoisomerase I inhibitors such as irinotecan have shown activity in the treatment of gastric cancer, with single agent irinotecan demonstrating a response rate of 23 %. Fluorouracil, which has been examined most extensively, produces a response rate of approximately 20 % [19]. However, complete responses with single agents are rare and partial regressions have been relatively brief. In addition, reduction in toxicity and better tolerance are important for gastric cancer patients. This “window of opportunity” phase II study was performed to evaluate the antitumour activity of TP300 in patients with advanced gastric or oesophago-gastric junction adenocarcinoma, receiving first-line chemotherapy without exposure to prior systemic chemotherapy for advanced disease. This study was a modified Simon’s two stage design [20].

## Methods

### Study design

Open-label, non-randomised, multi-centre study to evaluate the anti-tumour activity of TP300 in previously untreated patients with advanced gastric and/or gastro-oesophageal junction adenocarcinoma. The study complied with Good Clinical Practice and the Declaration of Helsinki, and was approved by research ethics committees in all institutions prior to initiation. All patients gave written informed consent for the clinical study and separately for the genotype/biomarker study before undergoing any study-related procedures.

The primary objective of this study was to determine the objective response rate (ORR) of TP300 in patients with untreated advanced gastric or gastro-oesophageal junction adenocarcinoma. The secondary objectives were to determine the time to progression (TTP) and progression free survival (PFS), to determine the safety of TP300 and to further evaluate the PK profile of TP300 evaluating any PK/PD correlation.

### Patients and eligibility criteria

Eligible patients had metastatic or locally advanced gastric or Siewert Types II or III GOJ inoperable adenocarcinoma. The patients were previously untreated with chemotherapy unless this had been administered in the perioperative setting, were ≥ 18 years old, with one or more measurable target lesions as defined by RECIST version 1.1, and had an Eastern Cooperative Oncology Group performance status of 1 or less.

Other inclusion criteria included adequate bone marrow function (neutrophil count ≥ 1.0 × 10^9^, platelet count ≥ 100 × 10^9^, and haemoglobin ≥9 g/dL) and adequate hepatic (serum bilirubin ≤ 1.5 x upper limit of normal [ULN], alanine amino transferase (ALT) and aspartate amino transferase (AST) ≤ 2.5 x ULN) and renal (serum creatinine ≤ 1.5 x ULN) function.

A history of severe or life-threatening drug allergy or hypersensitivity to camptothecin derivatives and diarrhoea (excess of 2-3 stools/day above normal frequency within 2 weeks prior to the start of the study) were exclusion criteria.

### Treatment and dose escalation

TP300 was administered as a 1-h intravenous (i.v.) infusion every 3 weeks (one cycle), for up to 6 cycles. The starting dose of TP300 was 8 mg/m^2^ in cycle 1. From cycle 2 onwards the dose was increased, where possible, to 10 mg/m^2^ in the absence of DLT but for some patients the dose was not changed or was lowered. This was to minimize the risk of serious toxicity. At the discretion of the investigator the treatment could be continued beyond cycle 6 if in the best interests of the patient.

### Evaluation of efficacy

Patients who received at least 2 cycles of TP300 and had at least 1 post-baseline tumour assessment were evaluable for assessment of response. After the baseline evaluation, tumours were assessed every 2 cycles using the Response Evaluation Criteria in Solid Tumors (RECIST) version 1.1. Responses were assessed by local investigators and by an independent radiologic review committee (IRC).

### Evaluation of toxicity

Toxicity was assessed weekly and graded using the National Cancer Institute Common Toxicity Criteria (CTCAE) version 3.0. DLT was defined as the occurrence of any of the following adverse events: grade 4 thrombocytopenia; febrile neutropenia or grade 4 neutropenia > 5 days duration; grade 4 diarrhoea not reduced to grade 1 within 2 days of appropriate therapy; other gastro-intestinal toxicities (e.g. vomiting, nausea, stomatitis) ≥ grade 3 and not reduced to grade 1 within 2 days of appropriate therapy; any other non-hematologic toxicities ≥ grade 3 (excluding alopecia).

### Statistical analysis

An optimal two-stage design was used where the estimated response rate was 25 % and the null hypothesis response rate was 10 % or less [20]. The study was calculated to have a one-sided α-error of 5 % and a power of 80 %. Under these assumptions, 18 evaluable patients were to be treated in stage 1 of the study. At least three responses were required to continue to stage 2 where an additional 25 evaluable patients would be treated (up to a total of 43). Overall, if a total of eight responses or more were observed, the drug would be considered active.

### Evaluation of pharmacokinetics

Blood samples for PK analysis were collected from each subject at 4 time points (pre dose, then 1, 5, and 24 h after the start of drug administration) in cycles 1 and 2. In brief, samples were processed by extraction of protein using addition of organic solvent containing internal standards. Samples were then directly injected into an LC-MS/MS system (Ionization: ESI positive, Mode: MRM). The lower limits of quantification of TP3076 and TP3011 in human plasma were 40 pg/mL, with the precisions being 7.1 to 12.1 % and 8.0 to 11.4 %, respectively.

## Results

### Patient characteristics

Twenty patients were enrolled into the study from 6 UK Centres between October 2009 and November 2010. One patient did not receive the study medication because of out of range pre-first dose blood results. Nineteen patients received the study medication, but one patient did not complete 2 cycles of treatment and could not be evaluated for tumour response. Eighteen patients received at least 2 cycles of treatment and were evaluable for tumour response.

Two of the 18 patients were deemed non-evaluable by the IRC due to the absence of an evaluable target lesion. Therefore 16 patients were included in the efficacy evaluation. Of 16 evaluable patients, 6 completed 2 cycles, 2 received 3 cycles, 3 received 4 cycles and 5 patients completed 6 cycles of treatment. Five patients did not receive dose escalation from 8 mg/m^2^ at cycle 1 to 10 mg/m^2^ in subsequent cycles. The median number of treatment cycles was 3. A summary of patient demographic characteristics is given in Table 1.

**Table 1:** Demographic and baseline characteristics (Safety Population)

	TP300
	*N* = 19
Sex	
Male	13 (68 %)
Female	6 (32 %)
Race	
Caucasian	19 (100 %)
Age in years	
Mean	66.1
SD	9.26
Median	67.0
Min-Max	40–82
Age (years)	
< 65	7 (37 %)
> =65	12 (63 %)
Duration from First Diag. to Dose in Days	
Median	53.0
Min-Max	33–3648
Diagnosis	
Gastric Adenocarcinoma	13 (68 %)
Gastro-oesophageal Junction Adenocarcinoma: Type II	4 (21 %)
Gastro-oesophageal Junction Adenocarcinoma: Type II	2 (11 %)
ECOG Performance Status	
0	12 (63 %)
1	7 (37 %)
Prior Surgery	
No	13 (68 %)
Yes	6 (32 %)
Prior Radiotherapy	
No	19 (100 %)
Prior Neoadjuvant Chemotherapy	
No	17 (89 %)
Yes	2 (11 %)
Prior Adjuvant Chemotherapy	
No	16 (84 %)
Yes	3 (16 %)

### Pharmacokinetics

Plasma mean (S.D.) drug concentration of unchanged drug and active metabolite in cycles 1 and 2 at 24 h after administration were 1.82 (5.85), 0.809 (0.635) and 5.39 ng/mL, respectively. The pharmacokinetics were similar to that established in the Phase I study [9].

### Efficacy

The tumour assessments were performed by investigative sites and the IRC. Although there were some differences between the two assessments, these were deemed within acceptable limits and the efficacy analysis was based on the IRC assessment.

**Fig. 2:**
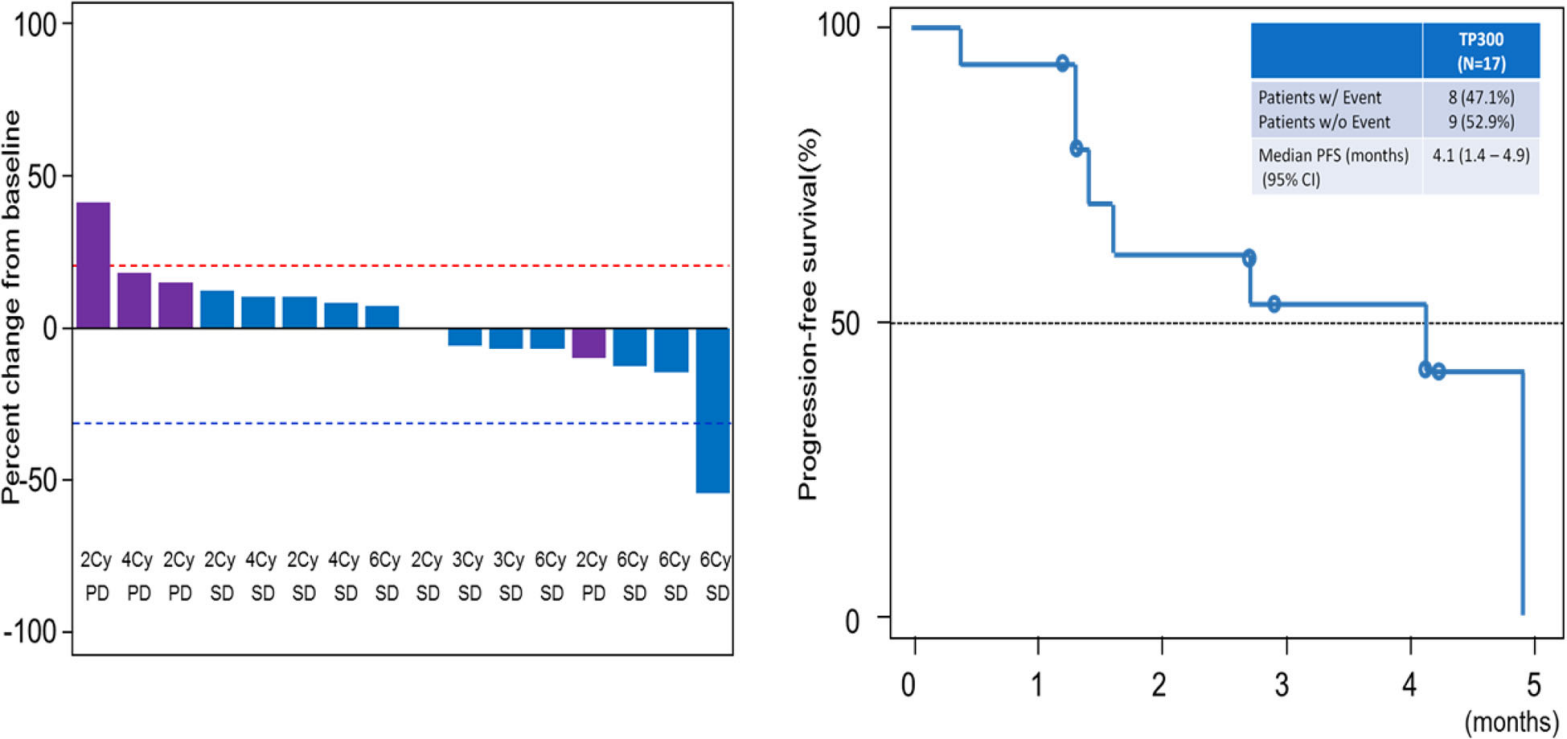
Waterfall plot and Kaplan Meier curve of PFS. Waterfall plot shows the 16 evaluable patients. 1 patient which showed a change from baseline greater than 50 %, was not a confirmed PR because there was no post end of treatment tumour assessment, and the best achieved response based on the first 2 cyclesconfirmed by IRC was SD

Of the 16 evaluable patients, no patients had a complete or partial response, 12 had stable disease, as their best response, for 1-5 months, and the other 4 patients had progressive disease after cycle 2. A summary of the best overall response is shown in Table 2. Eight events of progression, including one death occurred, and the median PFS was 4.1 months (CI [1.6–4.9 and the median TTP was 2.9 months (CI [1.4–4.2]) (Fig. 2).

**Table 2:** Summary of Best Overall Response (CR + PR) by Study Treatment (Assessed by Committee)

Evaluable patients	17
Complete Response	0
Partial Response	0
Stable Disease	12
Disease Progression	4
Not Evaluable	1
Overall Response Rate	0
Disease Control Rate	70.6 %

Evaluable Patients: The full analysis set (FAS) of 17 includes patients who received at least one dose of TP300 and had at least one measurable target-lesion confirmed by independent review committee (IRC)

Not Evaluable: 1 patient did not have a post dose tumour radiological assessment, so could not be assessed

Note: A further 2 patients who received TP300 were excluded from the FAS, as the target lesion/s could not be confirmed by IRC

### Toxicity

Diarrhoea, if it occurred, was grade 1-2 and of short duration. Grade 3-4 haematological toxicity was observed at a frequency similar to that of other cytotoxic agents with similar mode of action.

There was one case of gastrointestinal perforation, associated with a toxic death (grade 5). Overall there were 26 drug - related grade 3-4 AEs. Four patients experienced grade 4, and three patients grade 3 neutropenia. One patient experienced grade 3 and one patient grade 4 throbmbocytopaenia. One patient had grade 3 neutropenic sepsis which lasted 4 days and the dose of TP300 was subsequently reduced to 6 mg/m^2^. All haematological toxicities resolved. Among the grade 3-4 non-haematological toxicities, fatigue was the commonest (3 events of grade 3 intensity). There were 3 related grade 3-5 gastrointestinal events. The events of abdominal pain (grade 4), distension (grade 3) and perforation (grade 5) occurred in the same patient who had only received 1 cycle of 8 mg/m^2^.

Out of 10 DLTs, 3 occurred at doses less than 10 mg/m^2^; 2 of these were　thrombocytopenia grade 4 and neutropenia grade 4 in patients treated with 8 mg/m^2^. In both cases the dose was reduced to 6 mg/m^2^ without recurrence of the toxicity. The other DLT, at 8 mg/m^2^, occurred in a patient who received one dose of TP300 before presenting with a serious adverse event of gastrointestinal perforation with a fatal outcome. This event was deemed to be of low possible causality in relation to study drug TP300, since this patient had a pre-existing gastric ulcer. The remaining 7 DLTs, all at the 10 mg/m^2^ dose level, were grade 3 or 4 in intensity and except for the fatigue (which remained unresolved), resolved with either minor or no sequelae. In all these patients, subsequent doses of TP300 were reduced for the rest of the treatment cycles.

## Discussion

The dose chosen for this proof of concept study was the MTD in the Phase I study – on a three-weekly administration schedule. Although the MTD was defined at 10 mg/m^2^, and this was tolerable, the frequency of grade 3 neutropenia at this dose suggested it would be safer for patients to start on a dose of 8 mg/m^2^ for one cycle before escalating to 10 mg/m^2^ if well-tolerated. Four patients remained on 8 mg/m^2^, with no dose escalation, and 2 had a reduction to a lower dose after cycle 1, but overall it was considered that this was the correct dosing strategy.

The safety profile seen in the study was as anticipated from the phase I study. A significant number of patients developed haematological toxicity (37 % with grade 3 or 4 neutropenia), suggesting adequate dosing. Furthermore population PK-PD modelling from the phase I study indicated that the decrease in ANC following treatment with TP300 fitted well with total concentrations of TP3076 and TP3011 [21].

Other toxicities were similar to other topoisomerase I inhibitors (alopecia, fatigue etc.), with the exception of diarrhoea, which occurred less frequently than might have been expected with treatment using 3 weekly irinotecan. This is in keeping with previous clinical and pre-clinical studies of TP300.

Oesophago-gastric cancer response rates to single agent cytotoxic first-line are around 18 % [3] and patients were carefully chosen, and monitored on this study of an untried drug when a standard and reasonably effective combination treatment already existed. Hence the selection criteria for study entry focussed on those with a good performance status, low tumour burden and a normal serum albumin (a reliable prognostic factor), so that they were able to receive standard chemotherapy subsequently on disease progression.

The study design, with optimum dosing determined by tolerance of a dose lower than the Phase I defined MTD in the first cycle is unlikely to have led to suboptimal dosing. The fall in neutrophil count during cycle 1 (at 8 mg/m^2^) would support this. The small number of patients evaluated may have reduced the likelihood of detecting a weak response. Irinotecan monotherapy has shown response rates of 20 % in chemotherapy naïve patients with metastatic gastric cancer [13]. According to the Simon’s two stage design, 3 or more responders out of 18 evaluable patients at the interim analysis were necessary to allow initiation of stage II of the study. However, as no responders were seen amongst the 16 evaluable patients in stage I of the study it was not considered necessary to recruit further patients to achieve the target 18, because it would not have been possible to achieve 3 responding patients.

Examining the radiological responses in more detail, we found that even among the patients with stable disease there was wide variation in behaviour of the target lesions with some certainly increasing in size during treatment. There is insufficient information to confirm whether the exposure to TP300 in the tumour was adequate. However, we observed adequate evidence of a pharmacodynamic effect since there was significant bone marrow toxicity. The three-weekly administration regimen used in Phase I was carried into this study and, based on equivalent data from irinotecan, may have been expected to produce a measurable anti-tumour effect. It may be, however, that more frequent dosing (e.g. weekly), albeit with lower doses, would have given more promising results. Based on the manageable nature of haematological effects and the relative infrequency of other serious adverse reactions, there may be scope for pursuing higher tumour exposure by more frequent dosing.

## Conclusions

In conclusion, while this proof of concept study of TP300 in patients with advanced gastric or gastro-oesophageal cancer showed acceptable drug tolerability and confirmed the stable pharmacokinetics established in Phase I, and while there were cases of disease stabilisation for up to the maximum 6 cycles, it did not achieve the required primary end-point efficacy, as measured by complete or partial radiological response.

## Abbreviations

AE: Adverse event
ALT: Alanine amino transferase
AST: Aspartate amino transferase
AUC: Area under the curve
BCRP: Breast cancer resistance protein
CES2: Caroboxylesterase 2
CTCAE: Common Toxicity Criteria
DLT: Dose-limiting toxicity
ECOG: Eastern Cooperative Oncology Group
GC: Gastric cancer
GOJ: Gastro-oesophageal junction cancer
IRC: Independent radiological review committee
IV: Intravenous
LC-MS/MS: Liquid chromatography-mass spectrometry
MTD: Maximum tolerated dose
PD: Pharmacodynamic
PFS: Progression free survival
PK: Pharmacokinetic
RECIST: Response evaluation criteria in solid tumours
SAE: Severe adverse event
SD: Standard deviation
Topo-1: Topoisomerase-I
TTP: Time to progression
ULN: Upper limit of normal.

## References

[1] Beretta GL, Perego P, Zunino F (2008). Targeting topoisomerase I: molecular mechanisms and cellular determinants of response to topoisomerase I inhibitors. Expert Opin Ther Targets.

[2] FDA (2005). United States Food and Drug Administration: Camptosar label.

[3] Wagener DJ, Verdonk HE, Dirix LY (1995). Phase II trial of CPT-11 in patients with advanced pancreatic cancer, an EORTC early clinical trials group study. Ann Oncol.

[4] Bouche O, Raoul JL, Bonnetain F (2004). Randomized multicenter phase II trial of a biweekly regimen of fluorouracil and leucovorin (LV5FU2), LV5FU2 plus cisplatin, or LV5FU2 plus irinotecan in patients with previously untreated metastatic gastric cancer: a Federation Francophone de Cancerologie Digestive Group Study--FFCD 9803. J Clin Oncol.

[5] Langer CJ (2003). The global role of irinotecan in the treatment of lung cancer: 2003 update. Oncology (Williston Park).

[6] Vredenburgh JJ, Desjardins A, Reardon DA, Friedman HS (2009). Experience with irinotecan for the treatment of malignant glioma. Neuro Oncol.

[7] FDA (2005). United States Food and Drug Administration: Invader UGT1A1 molecular assay 510(k) summary.

[8] Endo M, Miwa M, Ura M (2009). A water soluble prodrug of a novel camptothecin analog is efficacious against breast cancer resistance protein-expressing tumor xenografts. Cancer Chemother Pharmacol.

[9] Anthoney DA (2012). Phase I study of TP300 in patients with advanced solid tumors with pharmacokinetic, pharmacogenetic and pharmacodynamic analyses. BMC Cancer.

[10] Kim YH (2005). Chemotherapy for advanced gastric cancer: slow but further progress. Cancer Res Treat.

[11] Cuschieri A, Weeden S, Fielding J (1999). Patient survival after D1 and D2 resection for gastric cancer: long term results of the MRC randomised surgical trial. Surgical Co-operative Group. Br J Cancer.

[12] Boku N, Ohtsu A, Shimada Y (1999). Phase II study of a combination of irinotecan and cisplatin against metastatic gastric cancer. J Clin Oncol.

[13] Kohne CH, Catane R, Klein B (2003). Irinotecan is active in chemonaive patients with metastatic gastric cancer: a phase II multicentric trial. Br J Cancer.

[14] Ajani JA, Baker J, Pisters PW (2002). CPT-11 plus cisplatin in patients with advanced, untreated gastric or gastroesophageal junction carcinoma: results of a phase II study. Cancer.

[15] Ajani JA, Baker J, Pisters PW (2002). Irinotecan/ cisplatin /in advanced, treated gastric or gastroesophageal junction carcinoma. Oncology.

[16] Assersohn L, Brown G, Cunningham D (2004). Phase II study of irinotecan and 5-fluorouracil/Leucovorin in patients with primary refractory or relapsed advanced oesophageal and gastric carcinoma. Ann Oncol.

[17] Glimelius B, Ekstrom K, Hoffman K (1977). Randomized comparison between chemotherapy plus best supportive care with best supportive care in advanced gastric cancer. Ann Oncol.

[18] Bugat R (2003). Irinotecan in the treatment of gastric cancer. Ann Oncol.

[19] Comis RL, Carter SK (1974). A review of chemotherapy in gastric cancer. Cancer.

[20] Simon R (1989). Optimal two-stage designs for phase II clinical trials. Control Clin Trials.

[21] Tomohisa S, Satofumi I, Masaichi A (2013). Population pharmacokinetic–pharmacodynamic modelling and simulation of neutropenia induced by TP300, a novel topoisomerase I inhibitor. J Pharm Pharmacol.

